# MicroRNA-155 and Disease-Related Immunohistochemical Parameters in Cutaneous Melanoma

**DOI:** 10.3390/diagnostics13061205

**Published:** 2023-03-22

**Authors:** Manal S. Fawzy, Afaf T. Ibrahiem, Naglaa A. Bayomy, Amin K. Makhdoom, Khalid S. Alanazi, Abdulaziz M. Alanazi, Abdulaziz M. Mukhlef, Eman A. Toraih

**Affiliations:** 1 Department of Biochemistry, Faculty of Medicine, Northern Border University, Arar 91431, Saudi Arabia; 2 Department of Pathology, Faculty of Medicine, Northern Border University, Arar 91431, Saudi Arabia; afaf.ali@nbu.edu.sa; 3 Department of Anatomy, Faculty of Medicine, Northern Border University, Arar 91431, Saudi Arabia; najlaa.bayoumy@nbu.edu.sa; 4 Faculty of Medicine, Northern Border University, Arar 91431, Saudi Arabia; akm.nbu@gmail.com (A.K.M.); drkh.077@gmail.com (K.S.A.); abdulaziz0alanazi@gmail.com (A.M.A.); wlehamson@gmail.com (A.M.M.); 5 Division of Endocrine and Oncologic Surgery, Department of Surgery, School of Medicine, Tulane University, New Orleans, LA 70112, USA

**Keywords:** melanoma, miR-155, gene expression, Real-Time PCR

## Abstract

Cutaneous melanoma is a severe and life-threatening form of skin cancer with growing incidences. While novel interventions have improved prognoses for these patients, early diagnosis of targeted treatment remains the most effective approach. MicroRNAs have grown to good use as potential biomarkers for early detection and as targets for treatment. miR-155 is well-studied for its role in tumor cell survival and proliferation in various tissues, although its role in melanoma remains controversial. In silico data analysis was performed in the dbDEMC v.3 to identify differentially expressed miRNA. We validated gene targets in melanoma using TarBase v8.0 and miRPath v3.0 and determined protein-protein interactions of the target genes. One hundred forty patients (age range 21–90 years) with cutaneous melanoma who underwent resection were included. Molecular assessment using Real-Time RT-qPCR, clinicopathological associations, and a literature review for the different roles of miR-155 in melanoma were performed. Analysis of the dbDEMC reveals controversial findings. While there is evidence of upregulation of miR-155 in primary and metastatic melanoma samples, others suggest decreased expression in later-stage melanoma and cases with brain metastasis. miR-155 has been overexpressed in prior cases of melanoma and precancerous lesions, and it was found to be dysregulated when compared to benign nevi. While miR-155 expression was associated with favorable outcomes in some studies, others showed an association with metastasis. Patients with high levels of miR-155 also noted reduction after receiving anti-PD-1 treatment, correlated with more prolonged overall survival. In our patient’s cohort, 22.9% relapsed during treatment, and 45% developed recurrence, associated with factors such as lymph node infiltration, high mitotic index, and positive staining for CD117. Although overall analysis revealed miR-155 downregulation in melanoma specimens compared to non-cancer tissues, increased expression of miR-155 was associated with cases of superficial spreading melanoma subtype (*p* = 0.005) and any melanoma with a high mitotic rate (*p* = 0.010). The analysis did not identify optimum cutoff values to predict relapse, recurrence, or mortality. In conclusion, miR-155 could have, in part, a potential prognostic utility in cutaneous melanoma. Further mechanistic studies are required to unravel the multifunctional role of miR-155 in melanoma.

## 1. Introduction

Cutaneous melanoma is the most serious form of skin cancer and is the leading cause of death from skin diseases. An epidemiological evaluation of the global burden estimated that 325,000 new cases of melanoma associated with 57,000 deaths occurred in 2020, with an estimated increase to 510,000 new cases and 96,000 deaths by 2040 [1]. Significant geographical variations in incidence rates across countries and world regions have been observed [2]. Risk factors associated with the development of cutaneous melanoma include fair skin and hair, excessive sun exposure, a family history of melanoma, and the presence of numerous or atypical moles, among others [3]. Cutaneous melanoma is more common in men than in women and is most commonly diagnosed in individuals between the ages of 45 and 55 [4]. The incidence of melanoma is also higher among Caucasians than other ethnic groups [5]. Due to its high mortality rate, melanoma has been a focus of research and intensive investigation over recent decades. With the development of surgical practices, concurrent radiation, chemotherapy, targeted therapy, and immunotherapy have greatly improved the prognosis of melanoma patients [6]. Nevertheless, the most effective way to improve the survival of cancer patients lies in early diagnosis and targeted treatment [7]. Therefore, many researchers are devoted to discovering novel biomarkers to predict the diagnosis or prognosis of melanoma and guide precise clinical treatments [8,9].

As vital regulators of diverse biological processes in cancer, microRNAs (miRNAs) were regarded as emerging potential biomarkers for cancer detection, predicting clinical outcomes, survival, and treatment responses [10,11,12]. They are a family of short, evolutionary conserved, noncoding single-stranded RNAs (19–24 nucleotides) that master regulates gene expression at the post-transcriptional level by binding to a complementary sequence in target mRNAs [13]. Increasing evidence revealed that miRNAs are involved in various physiological and pathological processes, such as cell differentiation, proliferation and apoptosis, invasion, metastasis, and immune responses [14,15,16]. MiRNAs are aberrantly expressed in various tumor types and regulate tumor biology by acting as oncogenes or tumor suppressors [17,18,19,20].

Among the different microRNAs, miR-155 is a classic example of a multifunctional miRNA that plays essential roles in regulating inflammatory response [21], autoimmunity [22], and cancer progression [23,24]. A large-scale miRNome analysis revealed miR-155 to be one of the top candidate markers for cancer pathogenesis [25], suggesting that miR-155 may serve as a bridge between inflammation and cancer [24,26]. MiR-155 is encoded by the third exon of the MIR155 host gene (MIR155HG), formerly called the B-cell Integration Cluster (BIC), located on human chromosome 21q21 [27]. Through interacting with multiple targets, miR-155 regulates a wide range of cellular and physiological processes, including inflammation, cell proliferation, and apoptosis [27]. In cancer, miR-155 has been associated with the regulation of tumorigenesis, metastasis, and angiogenesis. In particular, miR-155 targets a range of genes involved in tumor growth and survival, such as cyclin-dependent kinase inhibitors, proapoptotic proteins, and cell cycle regulators [28,29]. Additionally, miR-155 is implicated in the modulation of the immune system. It is involved in the activation/differentiation of immune cells, including B cells, T cells, dendritic cells, natural killer cells, myeloid cells, and macrophages [30,31,32]. Therefore, its deregulation can lead to decreased levels of the immune response, which can further contribute to tumor development and progression [33]. Notably, a clinical trial of cobomarsen, an inhibitor of miR-155 (NCT02580552), has been designed to treat patients diagnosed with cutaneous T-cell lymphoma, chronic lymphocytic leukemia, diffuse large B-cell lymphoma, or adult T-cell leukemia/lymphoma. These findings indicated that miR-155 plays a central role in tumor progression and the tumor microenvironment.

Several studies have reported the aberrant expression and genetic alterations of miR-155 tied to the diagnosis and clinical outcomes of various tumors. miR-155 is upregulated in numerous types of cancer, such as breast cancer [34], prostate cancer [23], colorectal cancer [35,36], lymphoma and hematological malignancies [37], and non-small cell lung cancer [38], while it was downregulated in esophageal cancer [39] and gastric cancer [40]. However, its role in the prognosis of melanoma remains unclear, with controversial findings [41,42,43,44,45,46,47,48]. Investigation of the molecular pathogenesis of malignant melanoma is critical to improving the survival of patients. In the current study, we aimed to investigate the diagnostic and prognostic value of miR-155 in malignant melanoma patients and to unravel the putative mechanisms driving melanoma progression bioinformatically.

## 2. Materials and Methods

### 2.1. In Silico Data Analysis

#### 2.1.1. Identification of Differentially Expressed Melanoma-Related miRNAs

We identified differentially expressed melanoma-related miRNAs across different experiments in the database of Differentially Expressed MiRNAs in human Cancers (dbDEMC v.3) (https://www.biosino.org/dbDEMC/index) (last accessed on 30 January 2023) [49,50]. It is an integrated database for platforms of microarray or miRNA-seq in cancers of human and model organisms detected by high-throughput and low-throughput methods recruited from public repositories, including Gene Expression Omnibus (GEO), Sequence Read Archive (SRA), ArrayExpress, and The Cancer Genome Atlas (TCGA). Specifically, miR-155-5p was deregulated in 33 cancer types. The absolute value of fold change at 1.0 was set to be a significant cutoff.

#### 2.1.2. Evidence-Based Melanoma miRNA Regulon

Experimentally validated gene targets of miR-155-5p in melanoma KEGG pathways were identified in Diana Lab tools using TarBase v8.0 (https://dianalab.e-ce.uth.gr/html/diana/web/index.php?r=tarbasev8 (last accessed on 30 January 2023)) and miRPath v3.0 (https://dianalab.e-ce.uth.gr/html/mirpathv3/index.php?r=mirpath) (last accessed on 30 January 2023). Protein-protein interactions of target genes were determined in String v11.5 (https://string-db.org (last accessed on 30 January 2023)). Additionally, literature-based gene-miRNA interactions were analyzed in Ingenuity Pathway Analysis (IPA, Qiagen, Hilden, Germany) software to define putative miR-155-related gene targets driving melanogenesis.

### 2.2. In-House Clinical Validation

#### 2.2.1. Study Population

Patients with cutaneous melanoma who underwent surgical resection as primary treatment were included. They were recruited from the Pathology labs of Mansoura and Suez Canal University Hospitals, Egypt, during the period between 2002 to 2016. A total of 140 paired archived formalin-fixed paraffin-embedded specimens of malignant melanomas were included. Inclusion criteria included samples with available complete clinical and pathological data obtained from the archived medical records and no prior history of adjuvant therapy. Pathological data related to CD133, CD117, CCND1 immunohistochemical staining, and BRAF mutation/expression were collected from the medical records and/or from unpublished work in the same authors’ lab. The study was conducted following the Declaration of Helsinki and approved by the Institutional Review Board.

#### 2.2.2. Clinical and Pathological Assessment

Cutaneous melanoma was classified into four types, namely, superficial spreading melanoma, nodular melanoma, acral lentiginous melanoma, and desmoplastic melanoma. The histopathological assessment included (1) TNM stage; (2) Breslow thickness: a measure of tumor thickness and the degree of invasion into the skin, measured in millimeters; and (3) Clark level: range from Level I (superficial) to Level V (deep), with higher levels indicating more severe tumors. Level I lesions are confined to the epidermis (melanoma in situ), while Level V lesions have invaded the underlying structures, including the dermis, subcutaneous fat, lymph nodes, and even muscle or bone, (4) mitotic rate (<5 and ≥5 mitosis/high-power field), and (5) ulceration [51,52]. 

Primary outcomes were as follows: (1) relapse is when symptoms return after a period of improvement; (2) recurrence is when symptoms reappear after a period of remission; and (3) mortality due to any cause. Time-to-event endpoints were assessed: (a) relapse-free survival was defined as the time from treatment to the relapse (local, regional, or distant recurrence), (b) disease-free survival was determined as the time from treatment to locoregional recurrence, metastasis, or death (for any reason), and (c) overall survival time was estimated from the time of treatment to death for any reason [53]. 

#### 2.2.3. RNA Extraction and Real-Time Reverse Transcriptase-Quantitative Polymerase Chain Reaction (RT-qPCR)

Five sections 5 μm thick of formalin-fixed paraffin-embedded (FFPE) tissue specimens were used for total RNA extraction. One hundred and sixty microliters of deparaffinization solution (Cat#: 19093; Qiagen, Hilden, Germany) was applied for paraffin removal from the specimens. Total RNA, including microRNA extraction, was carried out using the Qiagen miRNeasy FFPE Kit (Cat# 217504; Qiagen, Hilden, Germany), according to the provided protocol [54]. The lysis solutions supplied by the vendor and the incubation conditions permit the reversal of the formaldehyde modification and cross-linking of RNA and prevent further RNA degradation induced by tissue fixation. Each sample was subjected to proteinase K digestion and DNase treatment to remove genomic DNA, including the small fragmented nucleic acids and concentration of purified RNA, followed by elution in a volume of about 15–30 μL. The purity and concentration of extracted RNA were evaluated by “NanoDrop ND-1000 spectrophotometer (NanoDrop Tech. Inc., Wilmington, DE, USA)” then stored at −80 °C until the time of PCR.

MicroRNA-155 expression was quantified by a two-step RT-qPCR technique. First, to obtain complementary DNA (cDNA), reverse transcription was performed using “TaqMan MicroRNA Reverse Transcription (RT) kit (P/N 4366596; Applied Biosystems, Foster City, CA, USA)” and miR-155 specific stem-loop primers (5×) or RNU6B endogenous control primers (Cat#: 001039) for normalization. T-Professional Basic, Biometra PCR System (Biometra, Goettingen, Germany), was used under the thermal-cycling conditions as follows: “annealing at 16 °C for 30 min, extension at 42 °C for 30 min, enzyme inactivation at 85 °C for 5 min, and then holding at 4 °C” [55]. Appropriate negative controls were included in each run.

The Real-Time PCR was performed following the “Minimum Information for Publication of Quantitative Real-Time PCR Experiments (MIQE)” guidelines [56]. The relative expression of miR-155 mature sequence: “UUAAUGCUAAUCGUGAUAGGGGU” was quantified using the TaqMan^®^ assay (Applied Biosystems, assay ID: 002623, Cat#: 4427975). The RNU6B (assay ID: 001093) was used as an endogenous control in PCR as it showed a uniform/stable expression in tissues with no significant differences between cancer and non-cancer samples. PCRs were carried out in a total volume of 20 μL as explained previously [57], using StepOne™ Real-Time PCR System (Applied Biosystems). The PCR cycling conditions were as follows: “95 °C for 3 min 40 cycles of 95 °C for 15 s and 60 °C for 60 s” [58,59].

About 20% of randomly selected study specimens were re-evaluated in separate runs to test the PCR analysis reproducibility, and the results reproduced very close values of the quantification cycle (Cq) with low standard deviations (SD > 2.0 was set as an outlier). The fold change of gene expression in each sample relative to the mean of control samples was calculated using the comparative Livak formula based on the Cq value as the following: gene expression = 2^−ΔΔCq^ where, ΔCq = Cq of target gene (miR-155) − Cq of the reference gene (NBU6), the ΔΔCq: ΔCq (Cancer samples) − ΔCq (non-cancer samples) [60].

#### 2.2.4. Statistical Analysis

Data analysis was performed using SPSSv.27.0. Data were presented as frequency and percentage or mean and standard deviation (SD). Paired tests were used to compare cancer and corresponding non-cancer samples. The *p*-value was significant if the ≤0.05. MiR-155 molecular profile was correlated with clinical, pathological, and survival data. Kaplan–Meier and Cox proportional hazard regression analyses were applied to assess different risk factors.

### 2.3. Literature Screening and Review for the Role of miR-155 in Melanoma

Prior publications on the association between miR-155-5p and melanoma were screened in (a) GeneCards Human Gene Database (www.genecards.org): an integrative database that provides comprehensive, user-friendly information on all annotated and predicted human genes, (b) NCBI (www.ncbi.nlm.nih.gov/): National Center for Biotechnology Information for biomedical and genomic information, and (c) ncRPheno (http://lilab2.sysu.edu.cn/ncrpheno/ncrpheno.html): a comprehensive database that provides experimentally supported associations between noncoding RNAs and disease phenotypes across 11 species [61]. All databases were accessed on 30 January 2023.

## 3. Results

### 3.1. Database Screening for Deregulation of miR-155 in Melanoma

#### 3.1.1. Deregulated miR-155 in Melanoma

In the dbDEMC database, 89 comparisons showed upregulation, and 29 showed downregulation of miR-155-5p in various cancer types (Supplementary Table S1). In particular, melanoma experiments showed controversial findings. GSE24996 showed upregulation of miR-155-5p in primary melanoma samples (*n* = 15) compared to benign nevi (*n* = 8) (logFC (fold change) = 3014, q = 0.005). In another experiment (GSE18509), metastatic melanoma samples (*n* = 8) exhibited two-fold higher levels than benign nevi (*n* = 8) (logFC = 2.15, q = 0.002). In contrast, miR-155-5p was expressed at a significantly lower level in cutaneous melanoma stage 3 (*n* = 27) versus stage 2 (*n* = 60) in “TCGA” dataset (logFC = −1.85, q = 0.001). Similarly, the plasma level of hsa (homo sapiens = human)-miR-155-5p (SRP262521) was downregulated (logFC = −1.34, q = 0.024) in melanoma patients with brain metastasis (*n* = 36) compared to normal controls (*n* = 96).

#### 3.1.2. Molecular Targets of miR-155 in Melanoma

**Figure 1:**
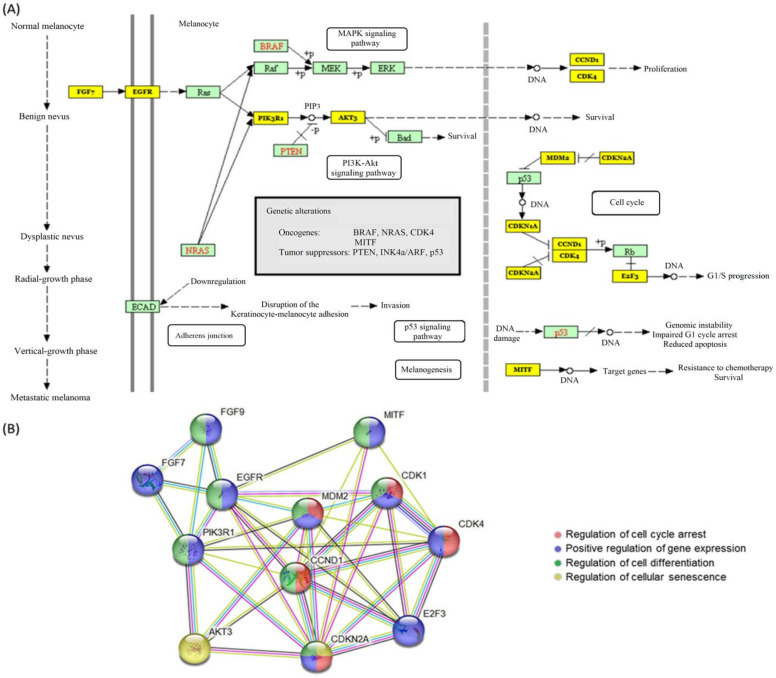
miR-155-5p gene targets in melanoma pathway. (**A**) Melanoma KEGG signaling pathway (hsa05218) is shown with the yellow box being experimentally validated gene targets for miR-155 [62]. Data source: Diana Lab Tools MirPath v3.0. (**B**) Melanoma targets protein-protein interaction network and functional enrichment analysis—data source: String version 11.5 (All accessed on 30 January 2023).

**Figure 2:**
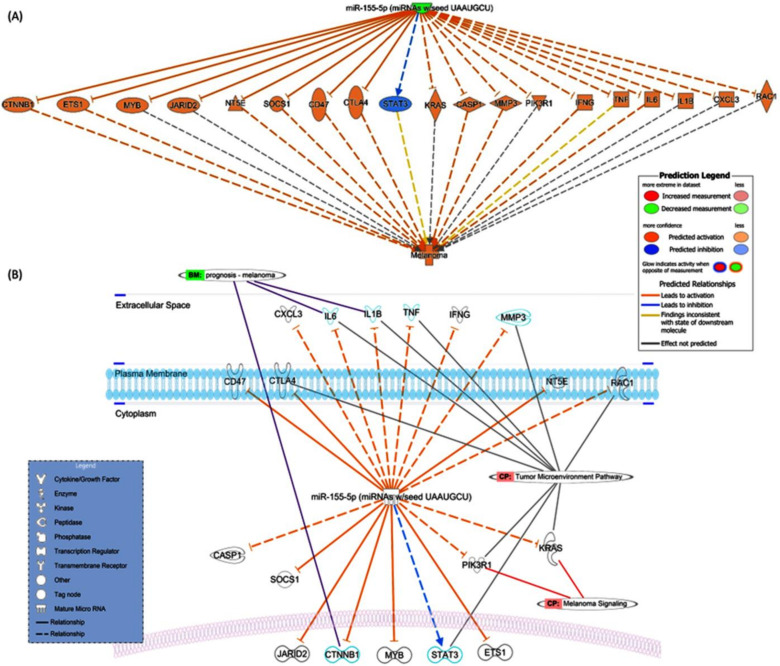
miRNA regulon in melanoma. (**A**) miR-155-5p and melanoma disease nodes were connected using the Path Explorer tool in the Ingenuity Pathway Analysis, based on published literature. Green node: downregulation, orange node: predicted activation, blue node: predicted inhibition. Edge: arrow with a line indicates activation, an arrow with a vertical line indicates inhibition, a solid line indicates direct interaction, dotted line indicates indirect interactions. (**B**) Cellular localization of miRNA targets displaying the type of molecular target and the evidence-based canonical pathway (CP) and biomarkers (BM).

As depicted in Figure 1A, miR-155-5p significantly targets 12 genes in the melanoma KEGG pathway [hsa05218] (*p* = 6.1 × 10^−34^), namely (1) *CDK4*, (2) *EGFR*, (3) *CDKN2A*, (4) *MITF*, (5) *CCND1*, (6) *E2F3*, (7) *PIK3R1*, (8) *FGF9*, (9) *AKT3*, (10) *CDKN1*, (11) *FGF7*, and (12) *MDM2* (Supplementary Table S2). Protein-protein interaction network revealed the role of these proteins in multiple cancer-related biological processes, including regulation of cell cycle arrest, gene expression, cell differentiation, and cellular senescence (Figure 1B). Using IPA software (Qiagen, Hilden, Germany), other novel melanoma-target genes were determined based on knowledge-based text mining (Figure 2).

### 3.2. Validation in Melanoma Cohorts

#### 3.2.1. Baseline Characteristics of In-House Patients

The study included 140 malignant melanoma patients. The mean age was 61.9 ± 14.9 years (ranged 21 to 90), the body mass index (BMI) was 26.6 ± 5.3 Kg/m^2^, and 54.3% were male. Notably, 20 patients had diabetes mellitus, 24 had hypertension, and 16 had hepatitis C. All lesions developed de novo; 45.7% appeared at the extremities, while 39.3% appeared in the trunk. Most lesions (75.7%) were nodular melanoma, 66.4% were ulcerating, 12% presented as multiple lesions at the initial diagnosis, and 5.7% were BRAF mutant samples (Table 1).

#### 3.2.2. Comparison between Indolent and Recurrent Cohorts

A total of 22.9% relapse during treatment, and 45% develop recurrence after cure. A comparison between indolent and progressive samples is depicted in Table 2. There was no significant difference regarding demographics or comorbidities; however, more frequency of clinical stage 3 was found (46% vs. 22.1%, *p* = 0.009). In addition, recurrent patients were more likely to have positive surgical margins following resection (49.2% vs. 11.7%, *p* < 0.001). Univariate regression analysis showed an odds ratio (OR) of 7.32 (95% CI = 3.12–17.2).

#### 3.2.3. Comparison between Survivors and Deceased Groups

Of the study population, 32.1% of cases died. Patients with residual margins (55.6% vs. 15.8%, *p* < 0.001) or who had received adjuvant therapy (33.3% vs. 13.7%, *p* = 0.012) had a higher risk of mortality (Table 3).

#### 3.2.4. Expression Pattern of miR-155 in Cancer Samples

The expression levels of miR-155 in melanoma tissue samples were lower than in paired non-cancer tissues. Median and interquartile range was −0.185 (IQR = −1.41 to 0.77), *p* = 0.050. As depicted in Table 4, tissue expression values of miR-155 were higher in superficial spreading melanoma (*p* = 0.005). Additionally, there were higher levels of miR-155 in samples with a high mitotic rate (*p* = 0.010). Receiver Operator Characteristics (ROC) curve analysis did not specify a significant optimum cutoff value to predict relapse (*p* = 0.84), recurrence (*p* = 0.88), or mortality (*p* = 0.99).

#### 3.2.5. Survival Analysis

**Figure 3:**
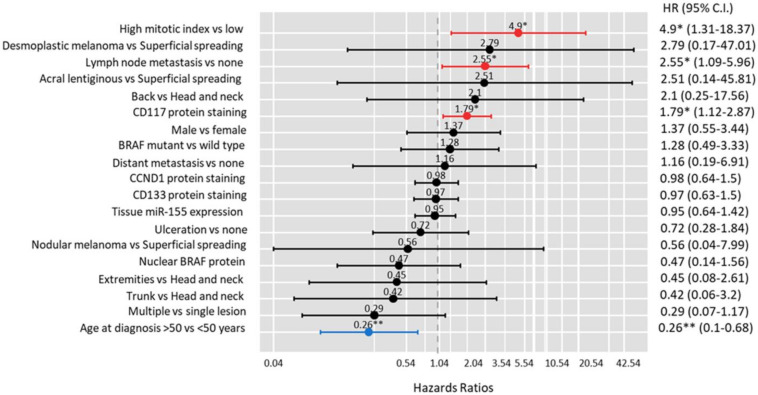
Risk factors predicting relapse in melanoma patients. Cox Proportionate Hazards regression analysis was performed. Results were reported as hazard ratio (HR) and 95% confidence interval (CI). The red bar indicates a significantly risky factor, while the blue bar indicates a protective one. Significance at * *p* < 0.05, ** *p* < 0.01.

After adjustment, melanoma patients presented lymph node infiltration (HR = 2.55, 95% CI = 1.09–5.96, *p* = 0.030), high mitotic index (4.90, 95% CI = 1.31–18.37, *p* = 0.018), and positive staining of CD117 protein (1.79, 95% CI = 1.12–2.87, *p* = 0.015) had a higher risk of relapse. In contrast, old patients (≥50 years at the time of diagnosis) were less likely to relapse (HR = 0.26, 95% CI = 0.10–0.68, *p* = 0.006) (Figure 3).

**Figure 4:**
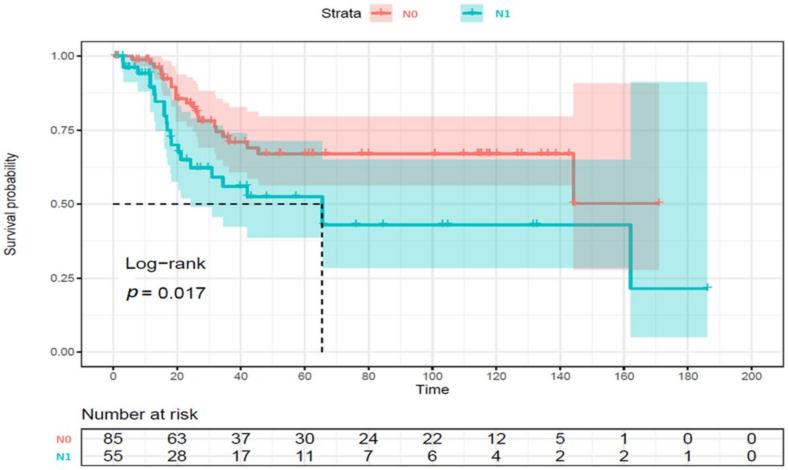
Kaplan–Meier curve for overall survival. The log-rank test was employed to compare patients with and without lymph node metastasis.

Multivariate Cox regression analysis showed that lymph node metastasis was a poor prognostic factor for recurrence and survival (Table 5). Kaplan–Meier survival curve for overall survival showed that patients with the N1 stage had shorter survival times (*p* = 0.017) (Figure 4).

## 4. Discussion

MiR-155 is an essential regulator of cancer biology and has been linked to the development and progression of malignant melanoma [26,63,64,65]. Similar to our findings that revealed miR-155 downregulation in melanoma tissues compared to non-cancer tissues, several studies have shown that miR-155 is under-expressed in melanoma [41,44]. Specifically, the loss of miR-155 expression has been linked to increased tumor aggressiveness, higher rates of metastasis, and increased resistance to chemotherapy drugs, as will be discussed in detail in the following subsections. MiR-155 appears to play an essential role in melanoma by suppressing the expression of various oncogenes, including Bcl-2, c-Myc, SMAD3, and PIK3CA, which are involved in cell proliferation, apoptosis, and angiogenesis, as shown in Figure 1 and Figure 2. In addition, miR-155 has been found to regulate the expression of genes involved in DNA repair, which may explain why patients in other studies with downregulated miR-155 were more likely to experience tumor recurrence [63,66]. 

### 4.1. Diagnostic Role of miR-155 in Human Subjects

In the present study, miR-155 showed significant downregulation in melanoma tissues relative to non-cancer tissues. Considering other coding and noncoding gene panels, this could help in the molecular profiling of this type of cancer, highlighting the potential role of miR-155 as a tissue-based marker that could have insight as a diagnostic molecular marker and future therapeutic target in melanoma, as supported by Poniewierska-Baran and colleagues in their interesting review [67].

A comparison of melanomas to benign nevi revealed that miR-155 was overexpressed in the malignant primaries [43,47]. Additionally, miR-155 levels were increased in Spitzoid melanomas in adults and children compared to benign nevi [48], and the expression was significantly higher in borderline melanocytic lesions [43]. Furthermore, miR-155 expression was higher in cases that had spread regionally [47]. Overexpression was found in metastatic tumor specimens obtained from stage IIIC–IV melanoma (subcutaneous, visceral, and LN metastases) [68]. It was also detected in human melanoma-derived exosomes [69], and elevated levels of exosomal miR-155 have been observed in the plasma of individuals with advanced melanoma (stage IIIC unresectable and stage IV) compared to healthy individuals [68,70]. The study of Lunavat et al. revealed that miR-155 was enriched in exosomes released by melanoma cells and was found to be deregulated in melanoma tissues and not in benign nevi [71].

### 4.2. Prognostic Role of miR-155 in Human Subjects

MiR-155 expression was linked to a favorable outcome in human melanoma patients and was associated with a robust immune signature in 29 other human solid tumors [33]. Higher expression of miR-155 has been linked to the outstanding production of cytokines and extended overall survival in melanoma patients [72]. However, other studies showed more elevation in cutaneous melanoma patients with a positive sentinel lymph node biopsy compared to those with a negative sentinel lymph node, and overexpression was associated with deeper invasion in both primary and metastatic melanomas [43,47]. These findings demonstrate that miR-155 strongly predicts melanoma progression and metastasis.

### 4.3. Expression Levels of miR-155 In Vitro and In Vivo

Arts et al. reported upregulation of miR-155 expression in four melanoma lines [73], providing evidence of a novel mechanism of immune escape in melanoma through miR-155 in an inflammatory microenvironment by targeting endogenous microphthalmia-associated transcription factor (MITF-M) in a mouse model. Nevertheless, in another study, miRNA-155 was found to be downregulated in the majority of melanoma cell lines (13 out of 17) [70]. Ectopic expression of miR-155 induced antiproliferative and proapoptotic effects in melanoma cells, suggesting that miRNA-155 may be a potential therapeutic target for melanoma [70]. Moreover, high expression of miR-155 was found to prolong the survival of cutaneous melanoma [33,74]. Furthermore, it has been demonstrated that miR-155 can target the *SKI* “a transcriptional-coregulator overexpressed in melanoma” gene in human melanoma cell lines, resulting in a growth-suppressive effect [75].

### 4.4. Potential Role of miR-155 in Melanoma Genesis

Several gene expression profiles of the normal human epidermal melanocytes and melanoma cells derived from primary/metastatic cells have been completed to obtain data on miRNA’s role in melanoma genesis [65,76,77]. 

While the diagnostic utility of miR-155 in melanoma has been established, its causal role in melanoma genesis is still being studied. However, there is evidence to suggest that miR-155 does play a role in melanoma development and progression [63,65]. 

Studies have shown that miR-155 expression is dysregulated in melanoma cells and could be associated with tumor growth and metastasis by targeting various genes involved in cell proliferation, apoptosis, and invasion. For instance, miR-155 has been found to target the tumor suppressor gene TP53, which is involved in regulating cell cycle progression and DNA repair [78]. Additionally, miR-155 has also been reported to target the apoptosis regulator BCL-6, which is involved in regulating cell survival and apoptosis [79]. Additionally, ectopic expression of miR-155 significantly inhibited the proliferation of melanoma cell lines and induced their apoptosis [41].

Furthermore, miR-155 has been implicated in the interaction between melanoma cells and the tumor microenvironment [26,33,72,80,81]. It has been shown to modulate the immune response by regulating the expression of cytokines and chemokines, which can affect the recruitment and function of immune cells in the tumor microenvironment, as will be detailed in the following subsections. 

### 4.5. Role of miR-155 in the Tumor Microenvironment of Melanoma Samples

#### 4.5.1. Role of miR-155 in Inflammation 

The role of miR-155 in regulating the immune cell dynamics of solid tumors has not been fully explored [33,79]. It has been demonstrated that miR-155 plays a central role in the inflammatory response and is an essential mediator for the interaction between cancer and inflammation [74,82]. miR-155 expression can produce inflammation in miR-146a-deficient mice by upregulating NF-κB activity [83]. Furthermore, inflammation mediated through JNK and NF-kB can regulate miR-155 expression and decreased expression of microphthalmia-associated transcription factor (MITF-M), which is a transcription factor that regulates the expression of genes associated with cytolytic T lymphocytes (CTL), leading to the immune escape of melanoma [73]. The effects of miR-155 on immune responses from various immune cells are outlined in Table 6, including their functions and target genes.

#### 4.5.2. miR-155 and T Cells

CD8+ T cells have been identified as a critical factor in antitumor activity and are thus a prime candidate for immunotherapy [91]. Studies have demonstrated that miRNAs can regulate pathways and molecules related to T cells activation [92], and miR-155 has been recognized as a critical modulator of the immune response [93], with high expression identified in T/B cells, dendritic cells, and macrophages [94]. MiR-155 overexpression in CD8+ T cells is linked to an increased antitumor response by reducing the expression of key molecules, such as signal transducer and activator of transcription 5 (STAT5), Src homology-2 domain-containing inositol 5-phosphatase 1 (SHIP1), Suppressor of cytokine signaling 1 (SOCS1), and Protein Tyrosine Phosphatase Non-Receptor Type 2 (PTPN2) [95]. Loss of miR-155 in T cells reduces antitumor immunity in a melanoma mouse model by decreasing the activated T-cell response and increasing the population of myeloid cells [33,85,86]. Moreover, miR-155 T-cell-conditional knockout mice exhibit enhanced tumor growth and reduced IFN-γ-expressing CD4+ and CD8+ T cells [85]. Furthermore, miR-155 acts as a suppressor of melanoma [75,96,97]. Immunotherapy may restore the antitumor activity caused by miR-155 deficiency in T cells by suppressing miR-155 target genes, such as SOCS1, BTB domain, and CNC homolog 1 (BACH1), CCAAT enhancer binding protein beta (CEBPB), and interleukin 7 receptor (IL7R) [85], suggesting that miR-155 levels may be used as a biomarker to determine the efficacy of immunotherapy.

#### 4.5.3. miR-155 and Cancer-Associated Fibroblasts (CAFs)

The introduction of exosomal miR-155 into human adult dermal fibroblasts has been demonstrated to modify the metabolic activities of the recipient cells, including increased glycolysis metabolism and less oxidative phosphorylation [69]. Additionally, melanoma cells have been reported to secrete miR-155-bearing exosomes that can differentiate the fibroblasts into proangiogenic Cancer-Associated Fibroblasts (CAFs), stimulating their proliferation, migration and tumor growth in vitro and in vivo [88]. This process was associated with activation of the JAK2/STAT3 signaling pathway and increased expression of matrix metallopeptidase 9 (MMP9), SOCS1, as well as proangiogenic cytokines, including vascular endothelial growth factor A (VEGFA), and fibroblast growth factor 2 (FGF2) [88].

#### 4.5.4. miR-155 and Macrophages

Studies have indicated that miRNAs can influence macrophage polarization, with miR-155 stimulating the activation of M1 macrophages. These M1 macrophages exhibit antitumor properties, while M2 macrophages encourage the growth of tumors. Tumor-associated macrophages (TAMs) have been linked to poor prognosis and survival in melanoma patients. MiR-155 has been identified as a regulator of the M1/M2 balance in human macrophages [87]. miR-155 achieves its activation of M2 macrophages through targeting SOCS1 [98], B-cell lymphoma-6 protein (BCL6) [90], and interleukin 13 receptor alpha 1 (IL13Rα1) [87], resulting in diminished activation of signal transducer and activator of transcription 6 (STAT6) [87]. Furthermore, miR-155 was downregulated in TAMs. Notably, miR-155/KD significantly accelerated tumor growth by impairing the classic activation of TAMs [99].

#### 4.5.5. miR-155 and Myeloid-Derived Suppressor Cells (MDSCs)

MicroRNAs (miRNAs), such as miR-155, were involved in the expansion of myeloid-derived suppressor cells (MDSCs), which are a heterogenous cell population composed of myeloid progenitor cells and immature myeloid cells. The miRNA target SHIP-1 to induce MDSCs [100] and SOCS1 to regulate their differentiation and accumulation [98]. Additionally, exosomal miR-155 is thought to be involved in the polarization and functions of MDSCs in melanoma [68].

#### 4.5.6. miR-155 and Natural Killer Cells (NK Cells)

The role of NK cells in tumor immunity has been studied, and various miRNAs have been found to affect their ability to suppress tumor growth by mediating the production of immunostimulatory cytokines. Investigations have shown that miR-155 is involved in the functions of NK cells in melanoma by positively regulating Killer Cell Lectin Like Receptor K1 (KLRK1), interferon (IFN-γ), and granzyme B production [31]. This is achieved by suppressing its negative regulator SH2 Domain-Containing Inositol 5′-Phosphatase 1 (SHIP1) [31]. 

#### 4.5.7. miR-155 and Tumor-Associated Mesenchymal Stem Cells (MSC)

MiRNA levels have been shown to influence the activation of Mesenchymal Stem Cells (MSCs) in tumor microenvironments (TME), promoting tumor growth and progression [65]. Zhu et al. demonstrated that downregulation of miR-155-5p causes activation of nuclear factor-κB p65 (NF-κB p65) in gastric cancer, while overexpression of miRNA suppresses the metastasis-promoting activity of cancer-associated MSCs in the prostate, multiple myeloma and myeloid neoplasms [101]. However, there is currently no evidence of miR-155 effects on MSCs in the TME of melanoma cancer.

### 4.6. Role of miR-155 in Predicting Treatment Response 

The use of checkpoint inhibitor immunotherapy (CII) through immune checkpoint inhibitors (ICIs), such as ipilimumab (cytotoxic T lymphocyte-associated antigen 4 (CTLA-4) target) and nivolumab and pembrolizumab (inhibitors of programmed cell death protein-1 (PD-1)), have revolutionized the treatment of metastatic melanoma. CII has proven more effective than BRAF-targeted therapy, though half of the patients fail to see long-lasting benefits. Research has found that miRNA expressions in the blood or tissues of melanoma patients were predictive of the outcome of immunotherapy, indicating that miRNAs could be used as practical and inexpensive biomarkers to assess the response rate or survival time of patients receiving such treatment. An increase in the expression of miR-155 after anti-PD-1 treatment was observed in vivo and in situ, accompanied by lower levels of PTPN2. Furthermore, in a study including 9 healthy donors and 13 melanomas, patients had high levels of miR-155, and reduced levels of miR-155 targets after receiving anti-PD-1 treatment were correlated with more prolonged overall survival [72]. 

A wealth of evidence suggests that high levels of MDSCs may be responsible for resistance to cancer immunotherapy [70]. MiR-155 was associated with expanding the MDSCs pool and resistance to treatment with immune checkpoint inhibitors in melanoma patients [68]. Similarly, in renal cell carcinoma, high expressions of miR-155 were detected in the non-responding patients to CII [102]. There were no available studies demonstrating the side effects of immune checkpoint immunotherapy.

Overall, our findings and previous data mining support that miR-155 could play dual functions in oncogenesis, acting as a tumor suppressor, for example, in melanoma, gastric cancer, and ovarian cancer, or an oncogene, such as in “breast cancer, colon cancer, pancreatic cancer, nasopharyngeal carcinoma, and oral squamous cell carcinoma” [103]. Lorusso et al. realized that “there are several strategies have emerged to utilize the role of miRNA to develop anticancer therapeutics” [104]; one of these strategies that could be applicable in our case is the use of synthetic miRNA mimics in patients with melanoma as replacement therapy to substitute the downregulated miR-155. In this sense, it is essential to understand the mechanistic functions of miR-155 in melanoma before considering its clinical applications. 

## 5. Conclusions

In summary, the present study revealed the downregulation of miR-155 in cutaneous melanoma compared to non-cancerous tissues. Although miR-155 expression does not show significant associations with most clinical and immunohistochemically detected protein expressions, increased expression of miR-155 was associated with superficial spreading melanoma subtype and any melanoma with a high mitotic rate.

## Figures and Tables

**Table 1:** Characteristics of melanoma cancer tissues.

Characteristic	Level	Value	Characteristic	Level	Value
**Demographics**			Mitotic rate	<5 mitosis/hpf	106 (75.7)
Age at diagnosis, y	Mean ± SD	61.9 ± 14.9		≥5 mitosis/hpf	34 (24.3)
Sex	Male	76 (54.3)	Breslow depth	Median (IQR)	7.0 (3.0–12)
BMI, Kg/m^2^	Mean ± SD		Clark level	I	23 (16.4)
**Clinical data**				II	28 (20)
Origin	De novo	140 (100)		III	13 (9.3)
Anatomic location	Head and neck	12 (8.6)		IV	39 (27.9)
	Extremities	64 (45.7)		V	37 (26.4)
	Back	9 (6.4)	**Molecular and IHC-related data**		
	Trunk	55 (39.3)	CD133 staining *	Negative	28 (20)
Multiple lesions at presentation	Negative	123 (87.9)		Weak intensity	25 (17.9)
	Positive	17 (12.1)		Moderate	37 (26.4)
Subtype	Superficial spreading	3 (2.1)		Strong intensity	50 (35.7)
	Acral lentiginous	25 (17.9)	CD117 staining *	Negative	49 (35)
	Nodular	106 (75.7)		Weak intensity	46 (32.9)
	Desmoplastic	6 (4.3)		Moderate	21 (15)
Ulceration	Negative	47 (33.6)		Strong intensity	24 (17.1)
	Positive	93 (66.4)	CCND1 staining *	Negative	24 (17.1)
Clinical stage	I	3 (2.1)		Weak intensity	40 (28.6)
	IIA	3 (2.1)		Moderate	21 (15)
	IIB	21 (15.0)		Strong intensity	55 (39.3)
	IIC	52 (37.1)	BRAF^V600E^ *	Wild type	62 (44.3)
	IIIA	4 (2.9)		Mutant	78 (55.7)
	IIIB	20 (14.3)	BRAF Protein *	Overexpression	94 (67.1)
	IIIC	20 (14.3)	miR-155-5p	Median (IQR)	−0.2 (−1.4, −0.7)
	IIID	2 (1.4)	**Treatment**		
	IV	15 (10.7)	Surgical margin	Free	100 (71.4)
**Pathological data**				Residue	40 (28.6)
T stage	T1	1 (0.7)	Adjuvant therapy	Negative	112 (80)
	T2a	3 (2.1)		Positive	28 (20)
	T2b	4 (2.9)	Type of adjuvant therapy	CVD	7 (5.0)
	T3a	7 (5)		Interferon	14 (10.0)
	T3b	8 (5.7)		DTC	5 (3.6)
	T4a	31 (22.1)		Dacarbazine	1 (0.7)
	T4b	86 (61.4)		Sunitinib	1 (0.7)
N stage	N0	85 (60.7)		Radiotherapy	1 (0.7)
	N1a	9 (6.4)	**Follow-up**		
	N1b	9 (6.4)	Relapse	Negative	108 (77.1)
	N2a	5 (3.6)		Positive	32 (22.9)
	N2b	15 (10.7)	Recurrence or progression	Negative	77 (55.0)
	N3	17 (12.1)		Positive	63 (45.0)
M stage	M0	125 (89.3)	Died	Negative	95 (67.9)
	M1	15 (10.7)		Positive	45 (32.1)

IQR: interquartile range, SD: standard deviation, BMI: Body mass index, IHC: immunohistochemistry, CVD: polychemotherapy regimen (Cisplatin, Vinblastine, Dacarbazine), DTC: Dopachrome tautomerase. * Data collected from patients’ medical records and/or unpublished work.

**Table 2:** Comparison between patients with and without recurrence.

Variables	Levels	Remitted (*n* = 77)	Recurrence (*n* = 63)	*p*-Value
**Demographics**				
Age at diagnosis, years	Mean ± SD	62.1 ± 15.0	61.6 ± 15.0	0.88
Sex	Male	40 (51.9)	36 (57.1)	0.61
Body mass index, Kg/m^2^	Mean ± SD	26.5 ± 4.76	26.8 ± 6.14	0.82
Diabetes mellitus	Positive	13 (16.9)	7 (11.1)	0.47
Hypertension	Positive	14 (18.2)	10 (15.9)	0.83
Hepatitis C	Positive	10 (13)	6 (9.5)	0.60
**Clinical data**				
Anatomic location	Head and neck	5 (6.5)	7 (11.1)	0.61
	Extremities	35 (45.5)	29 (46)	
	Back	4 (5.2)	5 (7.9)	
	Trunk	33 (42.9)	22 (34.9)	
Multiple lesions	Positive	10 (13)	7 (11.1)	0.80
Subtype	Superficial spreading	2 (2.6)	1 (1.6)	0.39
	Acral lentiginous	10 (13)	15 (23.8)	
	Nodular melanoma	62 (80.5)	44 (69.8)	
	Desmoplastic	3 (3.9)	3 (4.8)	
Ulceration	Positive	49 (63.6)	44 (69.8)	0.48
Clinical stage	I	3 (3.9)	0 (0)	**0.009**
	II	46 (59.7)	30 (47.6)	
	III	17 (22.1)	29 (46)	
	IV	11 (14.3)	4 (6.3)	
**Pathological data**				
Tumor size	T1	1 (1.3)	0 (0)	0.40
	T2	5 (6.5)	2 (3.2)	
	T3	6 (7.8)	9 (14.3)	
	T4	65 (84.4)	52 (82.5)	
Lymph node metastasis	Positive	25 (32.5)	30 (47.6)	0.08
Distant metastasis	Positive	11 (14.3)	4 (6.3)	0.17
Mitotic rate	≥5 mitosis/hpf	17 (22.1)	17 (27)	0.56
Breslow depth	Median (IQR)	7.0 (2.5–11.5)	8.0 (4.0–13)	0.026
Clark level	I	11 (14.3)	12 (19)	0.59
	II	15 (19.5)	13 (20.6)	
	III	9 (11.7)	4 (6.3)	
	IV	24 (31.2)	15 (23.8)	
	V	18 (23.4)	19 (30.2)	
**Treatment**				
Surgical margin	Free	68 (88.3)	32 (50.8)	**<0.001**
	Residue	9 (11.7)	31 (49.2)	
Adjuvant therapy	Positive	11 (14.3)	17 (27)	0.09
**Follow-up**				
Died	Positive	0 (0.0)	45 (71.4)	**<0.001**

IQR: interquartile range, SD: standard deviation. Chi-square, Fisher’s Exact, Student’s *t*-tests, and Mann–Whitney U tests were used. Bold values indicate statistical significance at *p* < 0.05.

**Table 3:** Comparison between survived and expired patients.

Variables	Levels	Survived (*n* = 95)	Died (*n* = 45)	*p*-Value
**Demographics**				
Age at diagnosis, years	Mean ± SD	61.7 ± 14.8	62.2 ± 15.4	0.85
Sex	Male	48 (50.5)	28 (62.2)	0.21
BMI, Kg/m^2^	Mean ± SD	27.1 ± 5.17	25.5 ± 5.7	0.17
Diabetes mellitus	Positive	13 (13.7)	7 (15.6)	0.79
Hypertension	Positive	17 (17.9)	7 (15.6)	0.81
Hepatitis C	Positive	10 (10.5)	6 (13.3)	0.77
**Clinical data**				
Anatomic location	Head and neck	6 (6.3)	6 (13.3)	0.23
	Extremities	42 (44.2)	22 (48.9)	
	Back	5 (5.3)	4 (8.9)	
	Trunk	42 (44.2)	13 (28.9)	
Multiple lesions	Positive	10 (10.5)	7 (15.6)	0.41
Subtype	Superficial spreading	2 (2.1)	1 (2.2)	0.07
	Acral lentiginous	12 (12.6)	13 (28.9)	
	Nodular melanoma	78 (82.1)	28 (62.2)	
	Desmoplastic	3 (3.2)	3 (6.7)	
Ulceration	Positive	61 (64.2)	32 (71.1)	0.45
Clinical stage	I	3 (3.2)	0 (0)	0.14
	II	54 (56.8)	22 (48.9)	
	III	26 (27.4)	20 (44.4)	
	IV	12 (12.6)	3 (6.7)	
**Pathological data**				
Tumor size	T1	1 (1.1)	0 (0)	0.39
	T2	6 (6.3)	1 (2.2)	
	T3	8 (8.4)	7 (15.6)	
	T4	80 (84.2)	37 (82.2)	
LNM	Positive	33 (34.7)	22 (48.9)	0.14
Distant metastasis	Positive	12 (12.6)	3 (6.7)	0.38
Mitotic rate	≥5 mitosis/hpf	21 (22.1)	13 (28.9)	0.40
Breslow depth	Median (IQR)	7.0 (3.0–11.0)	8.0 (4.0–14.5)	0.26
Clark level	I	12 (12.6)	11 (24.4)	0.31
	II	18 (18.9)	10 (22.2)	
	III	11 (11.6)	2 (4.4)	
	IV	28 (29.5)	11 (24.4)	
	V	26 (27.4)	11 (24.4)	
**Treatment**				
Surgical margin	Free	80 (84.2)	20 (44.4)	**<0.001**
	Residue	15 (15.8)	25 (55.6)	
Adjuvant therapy	Positive	13 (13.7)	15 (33.3)	**0.012**
**Follow-up**				
Relapse	Positive	11 (11.6)	21 (46.7)	**<0.001**
Recurrence	Positive	18 (18.9)	45 (100)	**<0.001**

IQR: interquartile range, SD: standard deviation, BMI: Body mass index, LNM: lymph node metastasis Chi-square, Fisher’s Exact, Student’s *t*-tests, and Mann–Whitney U tests were used. Bold values indicate statistical significance at *p* < 0.05.

**Table 4:** Association between miR-155 expression level and clinicopathological outcomes.

Variables		Number	Values	*p*-Value
**Demographics**				
Age at diagnosis, years	<50 years	30	0.24 (−1.30–1.04)	0.26
	≥50 years	110	−0.26 (−1.46–0.68)	
Sex	Female	64	−0.16 (−1.45–0.97)	0.48
	Male	76	−0.21 (−1.34–0.73)	
Diabetes mellitus	Negative	120	−0.09 (−1.38–0.84)	
	Positive	20	−0.97 (−1.57–0.10)	
Hypertension	Negative	116	−0.17 (−1.36–0.78)	
	Positive	24	−0.97 (−1.50–0.64)	
Hepatitis C	Negative	124	−0.17 (−1.37–0.77)	
	Positive	16	−0.83 (−2.08–1.03)	
**Clinical data**				
Anatomic location	Head and neck	12	−0.17 (−1.50–1.14)	0.73
	Extremities	64	−0.23 (−1.33–0.78)	
	Back	9	−0.25 (−1.57–1.54)	
	Trunk	55	0.09 (−1.45–0.68)	
Multiple lesions	Negative	123	−0.25 (−1.48–0.77)	0.19
	Positive	17	0.65 (−0.79–1.06)	
Subtype	Superficial spreading	3	2.84 (1.07–0.00)	**0.005**
	Acral lentiginous	25	0.27 (−1.44–0.92)	
	Nodular melanoma	106	−0.41 (−1.45–0.66)	
	Desmoplastic	6	1.04 (−0.41–1.82)	
Ulceration	Negative	47	0.19 (−1.42–0.78)	0.20
	Positive	93	−0.29 (−1.42–0.77)	
Clinical stage	I	3	−0.82 (−0.83–0.00)	0.69
	II	76	0.05 (−1.51–0.87)	
	III	46	−0.52 (−1.37–0.61)	
	IV	15	0.42 (−1.24–0.78)	
**Pathological data**				
Tumor size	T1	1	−0.83 (−1.28–0.19)	0.65
	T2	7	−0.29 (−1.26–0.68)	
	T3	15	−0.13 (−1.50–0.80)	
	T4	117	−0.83 (−1.12–−0.03)	
LNM	Negative	85	0.10 (−1.51–0.91)	0.41
	Positive	55	−0.29 (−1.29–0.67)	
Distant metastasis	Negative	125	−0.25 (−1.47–0.77)	0.46
	Positive	15	0.42 (−1.24–0.78)	
Mitotic rate	<5 mitosis/hpf	106	−0.57 (−1.52–0.63)	**0.010**
	≥5 mitosis/hpf	34	0.66 (−0.41–1.15)	
Clark level	I	23	0.51 (−1.51–1.76)	0.18
	II	28	0.21 (−1.22–0.83)	
	III	13	−1.31 (−2.12–1.40)	
	IV	39	−0.12 (−1.48–0.48)	
	V	37	−0.29 (−1.42–0.64)	
**Treatment**				
Surgical margin	Free	100	−0.23 (−1.38–0.77)	0.95
	Residue	40	−0.15 (−1.51–0.81)	
**Molecular markers**				
CD133	Negative	28	−0.52 (−1.65–0.68)	0.37
	Weak	25	−0.83 (−1.38–0.46)	
	Moderate	37	−0.12 (−1.39–0.94)	
	Intense	50	0.18 (−1.31–0.80)	
CD117	Negative	49	−0.29 (−1.52–0.73)	0.52
	Weak	46	0.20 (−1.41–1.15)	
	Moderate	21	0.16 (−1.38–0.57)	
	Intense	24	−0.59 (−1.48–0.33)	
CCND1	Negative	24	−0.52 (−1.65–0.68)	0.87
	Weak	40	−0.83 (−1.38–0.46)	
	Moderate	21	−0.12 (−1.39–0.94)	
	Intense	55	0.18 (−1.31–0.80)	
BRAFV^600E^	Negative	62	−0.09 (−1.30–0.83)	0.32
	Positive	78	−0.26 (−1.49–0.70)	
Nuclear BRAF	Negative	97	−0.29 (−1.44–0.77)	0.55
	Positive	42	−0.01 (−1.41–0.81)	
**Follow-up**				
Relapse	Negative	108	−0.18 (−1.44–0.80)	0.88
	Positive	32	−0.21 (−1.38–0.68)	
Recurrence	Negative	77	−0.13 (−1.45–0.89)	0.89
	Positive	63	−0.26 (−1.36–0.68)	
Survival	Survived	95	−0.16 (−1.45–0.89)	0.98
	Deceased	45	−0.25 (−1.32–0.63)	

Mann–Whitney and Kruskal–Wallis tests were used. Bold values indicate significance at *p* < 0.05.

**Table 5:** Independent predictor factors associated with recurrence and mortality.

Risk Factors	Recurrence		Overall Survival	
	HR (95% CI)	*p*-Value	HR (95% CI)	*p*-Value
Age at diagnosis >50 vs. <50 years	0.90 (0.48–1.72)	0.76	0.87 (0.38–1.99)	0.75
Male vs. female	1.31 (0.72–2.38)	0.38	1.35 (0.66–2.79)	0.41
Extremities vs. Head and neck	0.48 (0.19–1.24)	0.13	0.49 (0.16–1.45)	0.20
Back vs. Head and neck	1.11 (0.28–4.46)	0.88	1.38 (0.29–6.57)	0.69
Trunk vs. Head and neck	0.46 (0.15–1.44)	0.18	0.48 (0.13–1.75)	0.26
Acral lentiginous vs. Superficial spreading	1.59 (0.14–18.47)	0.71	1.73 (0.13–23.44)	0.68
Nodular melanoma vs. Superficial spreading	1.27 (0.13–12.68)	0.84	0.55 (0.05–6.09)	0.63
Desmoplastic vs. Superficial spreading	0.82 (0.06–10.41)	0.88	0.91 (0.07–12.71)	0.95
Multiple vs. single lesion	0.81 (0.30–2.21)	0.68	0.66 (0.22–2.00)	0.46
Ulceration vs. none	1.23 (0.65–2.32)	0.53	1.02 (0.46–2.28)	0.95
Lymph node metastasis vs. none	2.29 (1.31–4.03)	**0.004**	2.10 (1.08–4.09)	**0.029**
Distant metastasis vs. none	1.48 (0.43–5.05)	0.53	0.71 (0.17–3.00)	0.64
High mitotic index vs. low	2.15 (0.96–4.80)	0.06	1.69 (0.61–4.67)	0.31
Tissue miR-155 expression	1.04 (0.83–1.31)	0.74	1.03 (0.79–1.34)	0.81
CD133 protein staining	0.89 (0.68–1.16)	0.39	0.92 (0.67–1.26)	0.60
CD117 protein staining	1.25 (0.94–1.65)	0.12	1.31 (0.92–1.87)	0.13
CCND1 protein staining	1.01 (0.79–1.30)	0.93	1.02 (0.75–1.40)	0.88
BRAF mutant vs. wild type	1.14 (0.61–2.13)	0.68	1.19 (0.57–2.48)	0.65
Nuclear BRAF protein	0.62 (0.29–1.31)	0.21	0.87 (0.37–2.03)	0.74

Cox Proportionate Hazards regression analysis was performed. Results were reported as hazard ratio (HR) and 95% confidence interval (CI). The bold value indicates that significance was set at *p* < 0.05.

**Table 6:** Functions and interaction of miR-155 in immune cells in relation to melanoma.

Direction	Downstream Aberration	Function	Experimental Design	References
↑ miR-155	Targets: STAT5, SHIP1, SOCS1, and PTPN2 ↓ Akt and Stat5 signaling ↓	Enhancing CD8+ T-cell antitumor responses	In melanoma cell lines and murine models	[33,72,74,84,85,86]
↑ miR-155	Glycolysis metabolism ↑ Oxidative phosphorylation ↓	Formation of CAFs	Only in melanoma cell lines	[87]
↑ miR-155	Direct target: SOCS1 ↓ JAK2/STAT3 signaling ↑ Proangiogenic factors: VEGFa, FGF2, and MMP9 ↑	Formation and the proangiogenic switch of CAFs	In melanoma cell lines and murine models	[88]
↑ miR-155	IFN-inducible genes ↑	Promoting the activation of M2 macrophages	In melanoma cell lines and murine models	[87,89,90]
↑ miR-155	STAT3 downstream signaling pathways ↓	Polarization and T-cell-inhibiting functions of MDSCs	In melanoma cell lines and murine models	[68]
↑ miR-155	IFN-c and granzyme B production and NKG2D expression ↑	Decreasing the sensitivity of melanoma cells to NK cells’ cytolysis	Only in melanoma cell lines	[31]

## Data Availability

All datasets presented in this study are included in the article/Supplementary Material.

## Supplementary Materials

The following supporting information can be downloaded at https://www.mdpi.com/article/10.3390/diagnostics13061205/s1, Table S1: The expression level of hsa-miR-155 in cancers. Table S2: Annotation of miR-155 gene targets in melanoma KEGG pathway.
