# Supplementary material for: MicroRNA-155 and Disease-Related Immunohistochemical Parameters in Cutaneous Melanoma
Source: Diagnostics (Basel). 2023 Mar 22;13(6):1205. doi: 10.3390/diagnostics13061205 (PMC10047208; doi:10.3390/diagnostics13061205)
Supplement: Supplementary file 1 [file diagnostics-13-01205-s001.zip › diagnostics-2299148-supplementary.pdf]

Supplementary Table S1. The expression level of hsa-miR-155 in cancers.

Cancer Subtype	Source ID	Design	logFC	Expression Status
Adrenocortical cancer	TCGA_ACC	High grade vs. low grade	1.58	UP
	GSE143385	Cancer vs. normal	0.78	UP
Biliary tract cancer	TCGA_CHOL	Cancer vs. normal	1.31	UP
Bladder cancer	TCGA_BLCA	Cancer vs. normal	0.99	UP
Brain cancer	GSE41032	Cancer vs. normal	3.11	UP
	GSE112264	Blood	2.83	UP
	GSE90603	Cancer vs. normal	2.75	UP
	GSE158284	Cancer vs. normal	2.65	UP
	GSE25631	Cancer vs. normal	2.28	UP
	GSE138764	Cancer vs. normal	2.24	UP
	GSE139031	Blood	1.94	UP
	GSE113740	Blood	1.47	UP
	GSE113486	Blood	1.42	UP
	GSE13030	Cancer vs. normal	1.36	UP
	SRP262521	Blood	0.76	UP
	GSE100775	Subtype1 vs. subtype2	-0.36	DOWN
	SRP262521	Blood	-1.41	DOWN
Breast cancer	ERP120483	Subtype1 vs. subtype2	10.35	UP
	SRP057497	Cancer vs. normal	3.09	UP
	SRP154583	Cancer vs. normal	2.71	UP
	SRP057497	Cancer vs. normal	2.7	UP
	GSE45666	Cancer vs. normal	2.27	UP
	SRP154583	Cancer vs. normal	2.1	UP
	GSE45666	Cancer vs. normal	2.07	UP
	SRP057497	Cancer vs. normal	1.95	UP
	GSE40525	Cancer vs. normal	1.97	UP
	GSE38167	Cancer vs. normal	1.47	UP
	GSE113740	Blood	1.32	UP
	GSE2564	High grade vs. low grade	1.29	UP
	GSE113486	Blood	1.25	UP
	GSE4589	Cancer vs. normal	1.22	UP
	GSE40525	Cancer vs. normal	1.22	UP
	TCGA_BRCA	Cancer vs. normal	1.14	UP
	GSE40525	Cancer vs. normal	1.07	UP
	TCGA_BRCA	Cancer vs. normal	1.07	UP
	GSE40525	Cancer vs. normal	1.05	UP
	GSE106817	Blood	0.99	UP
	GSE86278	Subtype1 vs. subtype2	0.83	UP
	GSE80038	Cancer vs. normal	0.76	UP
	GSE86995	Metastasis	0.72	UP
	GSE57897	Cancer vs. normal	0.55	UP
	GSE155467	Treatment	0.51	UP

	TCGA_BRCA	High grade vs. low grade	0.47	UP
	GSE22216	High grade vs. low grade	0.41	UP
	GSE57897	Cancer vs. normal	0.34	UP
	GSE22216	High grade vs. low grade	0.29	UP
	GSE19783	Subtype1 vs. substype2	-0.26	DOWN
	GSE22216	Subtype1 vs. substype2	-0.31	DOWN
	TCGA_BRCA	High grade vs. low grade	-0.7	DOWN
	SRP057497	Subtype1 vs. subtype2	-0.75	DOWN
	SRP057497	Subtype1 vs. subtype2	-1.14	DOWN
	ERP120483	Subtype1 vs. subtype2	-10.7	DOWN
Colon cancer	TCGA_COAD	Cancer vs. normal	1.99	UP
	TCGA_COAD	High grade vs. low grade	1.59	UP
	GSE49246	Cancer vs. normal	0.3	UP
	GSE59856	Blood	0.07	UP
	GSE49246	Cancer vs. normal	-0.23	DOWN
	GSE18392	Cancer vs. normal	-0.25	DOWN
	GSE18392	High grade vs. low grade	-0.4	DOWN
	GSE73487	Cancer vs. normal	-0.52	DOWN
	GSE18392	Cancer vs. normal	-0.68	DOWN
	TCGA_COAD	High grade vs. low grade	-2.66	DOWN
Colorectal cancer	TCGA_READ	High grade vs. low grade	3.38	UP
	GSE113486	Blood	1.88	UP
	GSE113740	Blood	1.79	UP
	TCGA_READ	Cancer vs. normal	1.78	UP
	GSE112264	Blood	1.39	UP
	GSE106817	Blood	1.15	UP
	GSE56350	Metastasis	1.13	UP
	TCGA_READ	High grade vs. low grade	0.95	UP
	GSE30454	Cancer vs. normal	0.85	UP
	GSE106817	Blood	0.56	UP
	GSE128446	Cancer vs. normal	0.28	UP
	GSE38389	Cancer vs. normal	-0.19	DOWN
	TCGA_READ	High grade vs. low grade	-3.38	DOWN
	SRP056261	Exosomes	-4.67	DOWN
Endometrial cancer	GSE35794	Cancer vs. normal	3.17	UP
	TCGA_UCEC	Cancer vs. normal	1.22	UP
Esophageal cancer	GSE97049	Cancer vs. normal	1.91	UP
	GSE122497	Blood	1.17	UP
	GSE113486	Blood	0.99	UP
	GSE114110	Cancer vs. normal	0.95	UP
	GSE106817	Blood	0.94	UP
	GSE122497	Blood	0.83	UP
	GSE6188	Cancer vs. normal	0.61	UP
	TCGA_ESCA	High grade vs. low grade	-0.82	DOWN
	TCGA_ESCA	Cancer vs. normal	-0.83	DOWN
Gallbladder carcinoma	GSE104165	Cancer vs. normal	0.42	UP

Gastric cancer	GSE23739	Cancer vs. normal	0.9	UP
	GSE26595	Cancer vs. normal	0.79	UP
	GSE106817	Blood	0.57	UP
	GSE93415	Cancer vs. normal	-0.52	DOWN
	TCGA_STAD	High grade vs. low grade	-0.83	DOWN
Gastrointestinal stromal tumor	GSE31741	High grade vs. low grade	0.92	UP
Head and neck cancer	SRP078325	Exosomes	4.83	UP
	GSE124566	Cancer vs. normal	1.81	UP
	SRP078325	Exosomes	1.49	UP
	TCGA_HNSC	High grade vs. low grade	0.61	UP
	GSE131166	Subtype1 vs. subtype2	0.54	UP
	TCGA_HNSC	Cancer vs. normal	0.5	UP
	TCGA_HNSC	High grade vs. low grade	-0.4	DOWN
	GSE33232	Cancer vs. normal	-0.7	DOWN
Hepatocellular carcinoma	GSE40744	Cancer vs. normal	2.01	UP
	GSE19980	Subtype1 vs. substype2	1.29	UP
	GSE106817	Blood	0.85	UP
	GSE59856	Blood	0.11	UP
	GSE6857	Cancer vs. normal	0.27	UP
	GSE32957	Cancer vs. normal	2.01	UP
	GSE10694	Cancer vs. normal	-0.44	DOWN
	TCGA_LIHC	High grade vs. low grade	-1.17	DOWN
Kidney cancer	TCGA_KIRC	Cancer vs. normal	3.39	UP
	GSE11016	Cancer vs. normal	2.56	UP
	GSE12105	Cancer vs. normal	2.28	UP
	TCGA_KIRP	Cancer vs. normal	1.76	UP
	GSE116251	Cancer vs. normal	1.71	UP
	GSE95385	Cancer vs. normal	1.42	UP
	TCGA_KICH	High grade vs. low grade	1.41	UP
	GSE47582	Cancer vs. normal	1.24	UP
	TCGA_KIRC	High grade vs. low grade	0.99	UP
	TCGA_KICH	Cancer vs. normal	0.75	UP
	TCGA_KIRC	High grade vs. low grade	0.67	UP
	TCGA_KIRC	High grade vs. low grade	0.65	UP
	GSE48137	Blood	-0.25	DOWN
	GSE33332	Cancer vs. normal	-3.19	DOWN
Larynx cancer	GSE69127	Subtype1 vs. subtype2	-1.51	DOWN
Leukemia	E_MTAB_1454	Blood	3.61	UP
	GSE51908	Cancer vs. normal	2.84	UP
	GSE31629	Cancer vs. normal	1.22	UP
	GSE2564	Subtype1 vs. substype2	1.07	UP
	E_MTAB_1454	Blood	0.89	UP
	GSE2399	Cancer vs. normal	-1.51	DOWN
Lung cancer	TCGA_LUAD	Cancer vs. normal	2.42	UP
	GSE135918	Cancer vs. normal	1.82	UP

	TCGA_LUAD	High grade vs. low grade	1.62	UP
	GSE113486	Blood	1.13	UP
	GSE63805	Cancer vs. normal	0.77	UP
	GSE106817	Blood	0.77	UP
	GSE74190	Cancer vs. normal	0.68	UP
	GSE15008	Cancer vs. normal	0.42	UP
	GSE135918	Cancer vs. normal	-0.13	DOWN
	GSE29135	Subtype1 vs. substype2	-0.29	DOWN
Lymphoma	GSE2399	Cancer vs. normal	4.43	UP
	GSE31377	Cancer vs. normal	2.62	UP
	GSE2399	Subtype1 vs. substype2	1.87	UP
	GSE139031	Blood	1.6	UP
	GSE2399	Cancer vs. normal	1.29	UP
	GSE2564	Subtype1 vs. substype2	-1.01	DOWN
	GSE31377	Cancer vs. normal	-1.14	DOWN
	GSE16558	Cancer vs. normal	-1.29	DOWN
	GSE2399	Subtype1 vs. substype2	-1.31	DOWN
	GSE37053	Subtype1 vs. substype2	-1.45	DOWN
	GSE2399	Subtype1 vs. substype2	-1.62	DOWN
	GSE29491	Cancer vs. normal	-1.89	DOWN
	GSE37053	Subtype1 vs. substype2	-2.66	DOWN
	GSE22420	Subtype1 vs. substype2	-3	DOWN
	GSE17498	Cancer vs. normal	-4.69	DOWN
Melanoma	GSE24996	Cancer vs. normal	3.14	UP
	GSE18509	Metastasis	2.15	UP
	GSE31568	Blood	0.77	UP
	SRP262521	Blood	-1.34	DOWN
	TCGA_SKCM	High grade vs. low grade	-1.85	DOWN
Mesothelioma	GSE40345	Cancer vs. normal	1.9	UP
Nasopharyngeal cancer	GSE32960	Cancer vs. normal	-0.8	DOWN
	GSE118613	Blood	-2.14	DOWN
Oral squamous cell carcinoma	GSE28100	Cancer vs. normal	1.61	UP
	GSE137865	Cancer vs. normal	1.34	UP
	GSE45238	Cancer vs. normal	0.92	UP
	GSE113956	Blood	-0.01	DOWN
	GSE82064	Cancer vs. normal	-1.61	DOWN
Ovarian cancer	GSE83693	Cancer vs. normal	1.3	UP
	GSE106817	Blood	1.01	UP
	GSE31801	Blood	-0.16	DOWN
Pancreatic cancer	GSE140719	Cancer vs. normal	4.48	UP
	GSE163031	Cancer vs. normal	2.34	UP
	GSE106817	Blood	1.5	UP
	GSE25820	Cancer vs. normal	1.42	UP
	GSE112264	Blood	1.34	UP
	GSE41369	Cancer vs. normal	1.29	UP
	TCGA_PAAD	High grade vs. low grade	1.03	UP

	GSE71533	Cancer vs. normal	-0.12	DOWN
	GSE37406	Treatment	-0.77	DOWN
	GSE74562	Treatment	-1.36	DOWN
	TCGA_PAAD	Cancer vs. normal	-2.37	DOWN
Prostate cancer	GSE21036	Cancer vs. normal	0.61	UP
	E_MTAB_3397	Cancer vs. normal	-0.26	DOWN
	GSE8126	Cancer vs. normal	-0.57	DOWN
	E_MTAB_408	Cancer vs. normal	-0.57	DOWN
Sarcoma	GSE31045	Cancer vs. normal	2.19	UP
	GSE106817	Blood	1.45	UP
	GSE113486	Blood	1.29	UP
	GSE106817	Blood	0.81	UP
	GSE65071	Blood	0.51	UP
	GSE55625	Cancer vs. normal	0.48	UP
Small intestinal neuroendocrine tumor	GSE103317	Metastasis	-0.6	DOWN
	GSE103317	Cancer vs. normal	-2	DOWN
Testicular cancer	TCGA_TGCT	High grade vs. low grade	-1.38	DOWN
Thyroid cancer	TCGA_THCA	High grade vs. low grade	1.66	UP
	GSE97070	Cancer vs. normal	-0.25	DOWN
	TCGA_THCA	High grade vs. low grade	-0.61	DOWN
Tonsil cancer	GSE75630	Cancer vs. normal	-0.31	DOWN

The absolute value of fold change at 1.0 was set to be a significant cutoff. The orange-highlighted values indicate significant upregulation, while the green-highlighted values indicate significant downregulation. Data source: dbDEMC v.3 (https://www.biosino.org/dbDEMC/), (last accessed on 30 January 2023).

Supplementary Table S2. Annotation of miR-155 gene targets in melanoma KEGG pathway.

Symbol	Gene	Function
CDK4	Cyclin-dependent kinase 4	a Ser/Thr-kinase component of cyclin D-CDK4 complexes that phosphorylate and inhibit members of the retinoblastoma (RB) protein family including RB1 and regulate the cell-cycle progression during G(1)/S transition
EGFR	Epidermal growth factor receptor	Receptor tyrosine kinase binding ligands of the EGF family and activating several signaling cascades to convert extracellular cues into appropriate cellular responses. Known ligands include EGF, TGFA/TGF-alpha, amphiregulin, epigen/EPGN, BTC/betacellulin, epiregulin/EREG and HBEGF/heparin-binding EGF. Ligand binding triggers receptor homo- and/or heterodimerization and autophosphorylation on key cytoplasmic residues. The phosphorylated receptor recruit adapter proteins like GRB2 which in turn activates complex downstream signaling cascades.
CDKN2A	Cyclin-dependent kinase inhibitor 2A	Acts as a negative regulator of the proliferation of normal cells by interacting strongly with CDK4 and CDK6. This inhibits their ability to interact with cyclins D and to phosphorylate the retinoblastoma protein
MITF	Microphthalmia-associated transcription factor	Transcription factor that regulates the expression of genes with essential roles in cell differentiation, proliferation and survival. Binds to M-boxes (5'-TCATGTG-3') and symmetrical DNA sequences (E-boxes) (5'-CACGTG-3') found in the promoters of target genes, such as BCL2 and tyrosinase (TYR). Plays an important role in melanocyte development by regulating the expression of tyrosinase (TYR) and tyrosinase-related protein 1 (TYRP1). Plays a critical role in the differentiation of various cell types, such as neural crest-derived melanocyte.
CCND1	G1/S-specific cyclin-D1	Regulatory component of the cyclin D1-CDK4 (DC) complex that phosphorylates and inhibits members of the retinoblastoma (RB) protein family including RB1 and regulates the cell-cycle during G(1)/S transition. Phosphorylation of RB1 allows dissociation of the transcription factor E2F from the RB/E2F complex and the subsequent transcription of E2F target genes which are responsible for the progression through the G(1) phase. Hypophosphorylates RB1 in early G(1) phase. Cyclin D-CDK4 complexes are major integrators of various mitogenenic and antimitogenic signals.
E2F3	Transcription factor E2F3	Transcription activator that binds DNA cooperatively with DP proteins through the E2 recognition site, 5'-TTTC[CG]CGC- 3' found in the promoter region of a number of genes whose products are involved in cell cycle regulation or in DNA replication. The DRTF1/E2F complex functions in the control of cell-cycle progression from G1 to S phase. E2F3 binds specifically to RB1 in a cell-cycle dependent manner.
PIK3R1	Phosphoinositide-3-kinase regulatory	Phosphatidylinositol 3-kinase regulatory subunit alpha; Binds to activated (phosphorylated) protein-Tyr kinases, through its SH2 domain, and acts as an adapter, mediating the association of the p110 catalytic unit to the plasma membrane. Necessary for the insulin-

	subunit alpha/beta/delta	stimulated increase in glucose uptake and glycogen synthesis in insulin-sensitive tissues. Plays an important role in signaling in response to FGFR1, FGFR2, FGFR3, FGFR4, KITLG/SCF, KIT, PDGFRA and PDGFRB. Likewise, plays a role in ITGB2 signaling.
FGF9	Fibroblast growth factor 9	Plays an important role in the regulation of embryonic development, cell proliferation, cell differentiation and cell migration. May have a role in glial cell growth and differentiation during development, gliosis during repair and regeneration of brain tissue after damage, differentiation and survival of neuronal cells, and growth stimulation of glial tumors; Belongs to the heparin-binding growth factors family
AKT3	RAC-gamma serine/threonine-protein kinase	AKT3 is one of 3 closely related serine/threonine- protein kinases (AKT1, AKT2 and AKT3) called the AKT kinase, and which regulate many processes including metabolism, proliferation, cell survival, growth and angiogenesis. This is mediated through serine and/or threonine phosphorylation of a range of downstream substrates. Over 100 substrate candidates have been reported so far, but for most of them, no isoform specificity has been reported. AKT3 is the least studied AKT isoform.
CDKN1	Cyclin-dependent kinase 1	Plays a key role in the control of the eukaryotic cell cycle by modulating the centrosome cycle as well as mitotic onset; promotes G2-M transition and regulates G1 progress and G1-S transition via association with multiple interphase cyclins. Required in higher cells for entry into S-phase and mitosis. Phosphorylates PARVA/actopaxin, APC, AMPH, APC, BARD1, Bcl- xL/BCL2L1, BRCA2, CALD1, CASP8, CDC7, CDC20, CDC25A, CDC25C, CC2D1A, CENPA, CSNK2 proteins/CKII, FZR1/CDH1, CDK7, CEBPB, CHAMP1, DMD/dystrophin, EEF1 proteins/EF-1, EZH2, KIF11/EG5, EGFR, FANCG, FOS, etc.
FGF7	Fibroblast growth factor 7	Plays an important role in the regulation of embryonic development, cell proliferation and cell differentiation. Required for normal branching morphogenesis. Growth factor active on keratinocytes. Possible major paracrine effector of normal epithelial cell proliferation; Belongs to the heparin-binding growth factors family
MDM2	E3 ubiquitin-protein ligase Mdm2	E3 ubiquitin-protein ligase that mediates ubiquitination of p53/TP53, leading to its degradation by the proteasome. Inhibits p53/TP53- and p73/TP73-mediated cell cycle arrest and apoptosis by binding its transcriptional activation domain. Also acts as a ubiquitin ligase E3 toward itself and ARRB1. Permits the nuclear export of p53/TP53. Promotes proteasome-dependent ubiquitin-independent degradation of retinoblastoma RB1 protein. Inhibits DAXX-mediated apoptosis by inducing its ubiquitination and degradation. Component of the TRIM28/KAP1-MDM2-p53/TP53
